# Cerebral vein thrombosis: clinical manifestation and diagnosis

**DOI:** 10.1186/1471-2377-11-69

**Published:** 2011-06-10

**Authors:** Christian Tanislav, Ralf Siekmann, Nicole Sieweke, Jens Allendörfer, Wolfgang Pabst, Manfred Kaps, Erwin Stolz

**Affiliations:** 1 Department of Neurology, Justus Liebig University, Giessen, Germany; 2 Department of Neuroradiology, Justus Liebig University, Giessen, Germany; 3 Department of Neurology, Neurologische Klinik Bad Salzhausen, Germany; 4 Institute for Biomedicine and Epidemiology, Justus Liebig University, Giessen, Germany

## Abstract

**Background:**

Cerebral venous thrombosis (CVT) is a disease with a wide spectrum of symptoms and severity. In this study we analysed the predictive value of clinical signs and symptoms and the contribution of D-dimer measurements for diagnosis.

**Methods:**

We evaluated consecutive patients admitted with suspected CVT receiving non-invasive imaging. Symptoms and symptom combination as well as D-dimer levels were evaluated regarding their diagnostic value.

**Results:**

239 patients were included in this study, 170 (71%) were females. In 39 patients (16%) a CVT was found. For identifying a CVT patients underwent either a venous CT-angiography or MR-angiography or both. No combination of symptoms either alone or together with the D-dimer measurements had a sensitivity and positive predictive value as well as negative predictive value and specificity high enough to serve as red flag. D-dimer testing produced rates of 9% false positive and of 24% false negative results. For D-dimer values a Receiver Operating Characteristic curve (ROC) and the area under the curve (AUC = 0.921; CI: 0.864 - 0.977) were calculated. An increase of sensitivity above 0.9 results in a relevant decrease in specificity; a sensitivity of 0.9 matches a specificity value of 0.9. This corresponds to a D-dimer cut-off level of 0.16 μg/ml.

**Conclusion:**

Imaging as performed by venous CT-angiography or MR-angiography has a 1 to 2 in 10 chance to detect CVT when typical symptoms are present. D-dimer measurements are of limited clinical value because of false positive and negative results.

## Background

Patients with cerebral venous thrombosis (CVT) present with a remarkably wide spectrum of signs and symptoms. Most common are headaches (> 80%), seizures (approximately 40%), hemiparesis (approximately 40%), altered consciousness (15-20%), and papilledema (20-30%) [1-3]. The relative frequency in cohort of patients with increasing headache without previous headaches history or headaches in combination with neurological deficits or seizures has been reported at approximately 10%, but has not yet been confirmed by other studies [4].

The non-specificity of most of the presenting symptoms of CVT poses major problems in an emergency room setting, because i.e. it might be difficult to screen every patient with headaches for CVT by magnetic resonance imaging (MRI). Current data on the symptomatology of CVT give no information on the predictive value of specific modes of presentation or their combination. Elevated D-dimers have been reported to have a high diagnostic sensitivity and specificity [4]. Yet, other investigations found considerable rates of false positive and negative results [5-10].

In this study we aimed therefore to investigate the relative frequency of CVT in a cohort of patients with clinical symptoms suggestive of this disease. Further the predictive value of different clinical signs and symptoms in patients suspected to suffer of CVT should be determined.

## Methods

### Patients

In a retrospective study we analyzed all consecutive patients admitted to our neurological department between August 2002 and November 2007 who were initially suspected of suffering of CVT and underwent either magnetic resonance imaging and venous magnet resonance angiography (MRA) or venous computed tomography angiography (CTA). Routinely T1-, T2-weighted images, FLAIR, DWI, and T2* sequences were carried out. Diagnostic criteria followed usual guidelines [11]. Readers were not blinded to the patient's clinical situation. Patients were initially identified based on an imaging data base. Symptoms which lead to the suspicion of an underlying CVT were identified retrospectively by reviewing case files. The medical history and neurological examination were analyzed regarding the findings which led to the suspicion of CVT. Papilledema was assessed clinically by ophthalmoscopy. All patients with suspected papilledema were examined by an ophthalmologist. Headaches were recorded as acute when onset was ≤ 24 hours, or as subacute when they lasted > 1 day. Further, thrombus localization and the presence of venous infarcts were registered. D-dimers in serum were measured using a turbidimetric method with a normal reference range below 0.19 μg/ml and were recorded when performed on the day of admission. The study protocol was reviewed and approved by the local ethical committee of the Justus Liebig University of Giessen.

### Statistical Evaluation

For defined parameters the positive predictive value (PPV), negative predictive value (NPV), sensitivity and specificity were calculated based on cross-tables. To compare nonparametric data the Mann-Whitney U-test was applied. For frequency data a χ^2^-test or when appropriate Fisher's exact test were used.

For the determination of different cut-off points for D-dimer levels indicating a CVT the true positive rate (sensitivity) in function of the false positive rate (1-specificity) was plotted to a Receiver Operating Characteristic (ROC) curve. For the estimation of the discriminatory accuracy of D-dimer measurement in indicating a CVT the area under the ROC curve (AUC) was calculated.

For identifying specific combinations of symptoms indicating a CVT, clusters were built applying the Schwarz's Bayesian criterion with logarithmic-likelihood distance measure.

## Results

In total, we identified 239 patients (170 [71%] females, 69 [29%] males) who received MRI and venous MRA (188 patients, 78.7%) or venous CTA (61 patients, 25.5%) to rule out CVT; in 10 patients both procedures were performed. None had conventional angiography. Important demographic data are summarized in Table 1.

**Table 1:** Demographic data

	CVT n = 39 (16.3%)	No CVT n = 200 (83.7%)	p
**Age (y)**	43.9	42.5	0.73
**Sex**			0.93
Males	12 (31%)	57 (29%)	
Females	27 (69%)	143 (71%)	
**Imaging**			
MRI/MRA	30 (77%)	158 (79%)	0.94
CTA	12 (31%)	49 (24%)	0.62
**Symptoms/History**			
Headaches	27 (69%)	145 (73%)	0.83
- acute (≤ 1day)	2 (5%)	34 (17%)	0.09
- subacte (> 1 day)	25 (64%)	111 (56%)	0.41
- unilateral	7 (18%)	30 (15%)	0.63
Seizure (focal or generalized)	11 (28%)	19 (10%)	**0.003**
Focal neurological deficit	17 (44%)	68 (34%)	0.33
Papilledema	4 (10%)	4 (4%)	0.15
Disturbed consciousness	0 (0%)	10 (5%)	0.32
Pregnancy*	3 (11%)	12 (8%)	0.43
Previous venous thrombosis	5 (13%)	24 (12%)	0.90
Oral contraceptives*	6 (22%)	29 (20%)	0.49
**D-Dimer measurement**	29 (74%)	69 (35%)	**0.001**
Increased^§^	22 (76%)	6 (9%)	**0.001**
Absolute values (μg/ml), median (range)	0.21 (0.09 - 2.51)	0.07 (0.01 - 3.1)	**0.001**
**Thrombus location**		Not applicable	
Superior sagittal sinus	14 (36%)		
Transverse sinus	24 (83%)		
Sigmoid sinus	6 (21%)		
Internal jugular vein	3 (10%)		
Straight sinus	10 (34%)		
Cortical veins	1 (3%)		

* limited to females.

^§ ^limited to patients with D-dimer measurements, reference range below 0.19 μg/ml.

**Figure 1:**
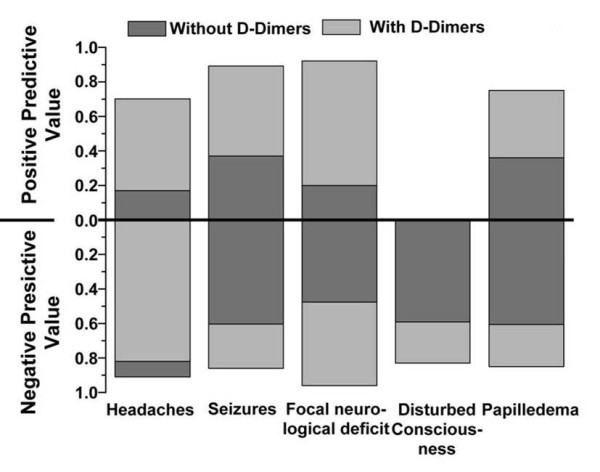
**Positive and negative predictive values for main symptoms with and without D-dimer measurements**.

**Figure 2:**
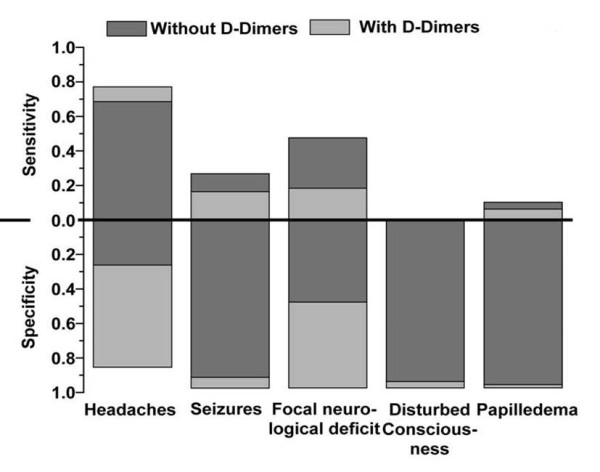
**Sensitivity and specificity for main symptoms with and without D-dimer measurements**.

We found CVT in 16.3% of patients. Presenting symptoms and thrombus location are listed in Table 1. Highest PPV was observed for the combinations of headaches plus seizures (0.57), headaches plus papilledema (0.57) and focal neurological deficit plus papilledema (1.0) (Table 2, Figures 1 and 2).

**Table 2:** Positive (PPV) and negative predictive value (NPV), sensitivity (Sens), specificity (Spec), and effectivity (Effect) of symptoms and symptom combinations in diagnosis of cerebral venous thrombosis (CVT)

Parameter	CVT vs. no CVT	PPV	NPV	Sens	Spec	Effect
**Headaches (any form)**	27/39 vs. 145/200	0.17	0.82	0.69	0.28	0.34
- plus seizure (focal or generalized)	4/39 vs. 3/200	0.57	0.85	0.10	0.99	0.84
- plus focal neurological deficit	12/39 vs. 39/200	0.23	0.86	0.31	0.81	0.72
- plus previous history of DVT or PE	5/39 vs. 24/200	0.33	0.84	0.07	0.97	0.82
- plus papilledema	4/39 vs. 3/200	0.57	0.85	0.10	0.99	0.84
- plus altered consciousness	0/39 vs. 1/200	0.00	0.84	0.00	0.99	0.83
- plus oral contraceptives*	6/27 vs. 26/143	0.19	0.85	0.22	0.82	0.72
- plus increased D-dimers§	14/29 vs. 7/69	0.67	0.81	0.48	0.89	0.78
**Seizures (focal or generalized)**	11/39 vs. 19/200	0.37	0.86	0.28	0.91	0.80
- plus focal neurological deficit	2/39 vs. 3/200	0.40	0.84	0.05	0.99	0.83
- plus previous history of DVT or PE	1/39 vs. 1/200	0.50	0.84	0.03	0.99	0.84
- plus papilledema	0/39 vs. 0/200	-	-	-	-	-
- plus altered consciousness	0/39 vs. 1/200	0.00	0.84	0.00	0.99	0.83
- plus oral contraceptives*	1/27 vs. 1/143	0.03	0.96	0.17	0.80	0.78
- plus increased D-dimers§	8/29 vs. 1/69	0.89	0.76	0.28	0.98	0.78
**Focal neurological deficit**	17/39 vs. 68/200	0.20	0.86	0.44	0.66	0.62
- plus previous history of DVT or PE	1/39 vs. 6/200	0.14	0.83	0.02	0.97	0.81
- plus papilledema	2/39 vs. 0/200	1.00	0.84	0.05	1.00	0.85
- plus altered consciousness	0/39 vs. 2/200	0.00	0.84	0.00	0.99	0.83
- plus oral contraceptives*	3/27 vs. 5/143	0.38	0.85	0.11	0.97	0.83
- plus increased D-dimers§	11/29 vs. 1/69	0.92	0.79	0.38	0.98	0.81
**Altered consciousness**	0/39 vs. 10/200	0.00	0.83	0.00	0.95	0.79
- plus previous history of DVT or PE	0/39 vs. 1/200	0.00	0.84	0.00	0.99	0.83
- plus papilledema	0/39 vs. 0/200	-	-	-	-	-
- plus oral contraceptives*	0/39 vs. 1/200	0.00	0.84	0.00	0.99	0.83
- plus increased D-dimers§	0/29 vs. 1/69	0.00	0.70	0.00	0.99	0.69
**Papilledema**	4/39 vs. 7/200	0.36	0.85	0.10	0.97	0.82
- plus previous history of DVT or PE	1/39 vs. 1/200	0.50	0.84	0.03	0.99	0.84
- plus oral contraceptives*	1/39 vs. 1/200	0.50	0.84	0.03	0.99	0.84
- plus increased D-dimers^§^	3/29 vs. 1/69	0.75	0.72	0.10	0.99	0.72
**Increased D-dimers§**	22/29 vs. 6/69	0.79	0.90	0.76	0.91	0.87

DVT: deep vein thrombosis of the legs

PE: pulmonary embolism

*limited to females

^§^limited to patients with D-dimer measurements

**Figure 3:**
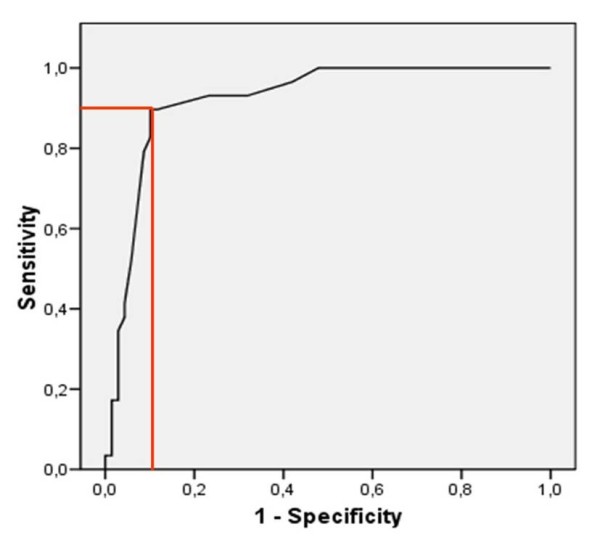
**ROC curve for diagnosing a cerebral vein thrombosis by D-dimer measurement**.

In 98 patients D-dimer levels were determined within 6 hours after admission. An increased D-dimer value was significantly more frequent in patients with CVT (p < 0.01); comparing absolute ranges higher values for D-dimers were detected in patients with CVT (median 0.21 μg/ml versus 0.07 μg/ml). D-dimer measurements primarily increased PPV and specificity (Figures 1 and 2). We found rates of 9% false positive and of 24% false negative results. For D-dimer values a ROC curve and the area under the curve (AUC = 0.921; CI: 0.864 - 0.977) were calculated (Figure 3). An increase of sensitivity above 0.9 results in a relevant decrease in specificity; a sensitivity of 0.9 matches a specificity value of 0.9 (Figure 3). The 90% percentile D-dimer value therefore corresponds to a cut-off level (0.16 μg/ml) for the identification of patients with CVT with a sensitivity of 90% and a specificity of 90%.

For identifying specific combinations of symptoms potentially indicating a CVT, 2 clusters were calculated. Considering redundancies the following parameters were selected: gender, seizure, focal neurological deficit, headache, papilledema, altered consciousness and previous DVT or PE. The quality of the clusters was slightly above average as indicated by the silhouette- cohesion- and separation coefficient (k = 0.4). The distribution of parameters among the 2 clusters is summarized in Table 3. Cluster 1 includes the majority of the patients with CVT (32/133 versus 7/106; p < 0.001); a sensitivity of 0.82 and a specificity of 0.50 respectively was calculated, respectively.

**Table 3:** Distribution of parameters among clusters

	Cluster 1 n = 133 (55.6%)	Cluster 2 n = 106 (44.4%)	*p*
**Sex**			0.06
Males	45 (34%)	24 (23%)	
Females	88 (66%)	82 (77%)	
**Symptoms**			
Headaches	66 (50%)	106 (100%)	< 0.001
Seizure (focal or generalized)	30 (23%)	0 (0%)	< 0.001
Focal neurological deficit	85 (64%)	0 (0%)	< 0.001
Papilledema	11 (8%)	0 (0%)	0.001
Disturbed consciousness	10 (8%)	0 (0%)	0.003
**Previous history of DVT or PE**	17 (13%)	0 (0%)	< 0.001
**CVT**	32 (24%)	7 (7%)	< 0.001

DVT: deep vein thrombosis of the legs

PE: pulmonary embolism

CVT: cerebral vein thrombosis.

## Discussion

The symptomatology of this patient cohort complies well with prospective CVT patients collectives [1,4]. We found an astonishingly high rate of CVT of approximately 16% in our patients which also corroborates results of a prospective study aimed at investigating the diagnostic value of D-dimers [4] which found a rate of approximately 10%.

No symptom combination reached a high enough PPV and sensitivity as well as NPV and specificity to serve as a red flag although headaches plus seizures, headaches plus papilledema, and focal neurological deficit plus papilledema (Table 2, Figures 1 and 2) are suggestive of CVT. When typical symptoms are present there is a 1 to 2 in 10 chance to detect CVT by appropriate non-invasive imaging. Even a cluster analysis did not identify a combination of symptoms with acceptable sensitivity and specificity in indicating a CVT.

In our cohort D-dimer testing produced rates of 9% false positive and of 24% false negative results which sheds serious doubts on their usefulness in a clinical setting. In cohorts with typical symptomatology they may primarily increase specificity. In contrast, the ROC analysis revealed a sensitivity of 90% and a specificity of 90% when considering a D-dimer cut-off value of 0.16 μg/ml; the AUC indicates a favorable discriminatory accuracy for D-dimer in indicating a CVT in general. However, considering normal ranges established in our laboratory (< 0.19 μg/ml) a value of 0.16 μg/ml might be regarded as normal. On the other hand, normal ranges for D-dimer values are derived considering venous embolic events such as pulmonary embolism or venous thrombosis of the pelvic and leg veins. Therefore a D-dimer measurement might have a beneficial impact, nonetheless the determined cut-of value of 0.16 μg/ml has to be regarded a pivotal finding, which requires verification.

A limitation of this investigation is its retrospective design. Missing patients are unlikely because the data base used is also the basis for hospital payment. In all cases the treating neurologist already considered CVT as potential differential diagnosis which may introduce a selection bias. Presumably this explains the relatively high percentage of CVT patients in our cohort. The higher rate of D-dimer measurements in CVT patients compared with non-CVT patients is most likely due to the fact, that treating neurologists were not blinded to the imaging results and diagnosis.

## Conclusions

Even considering limitations the results of this investigation clearly emphasizes that when typical symptoms are present a rate of 10 to 20% of CVT can be suspected. CVT is not a rare condition. There is no typical red flag symptomatology. The usefulness of D-dimer measurements is limited because measurements produce false positive and negative results.

## Abbreviations list

CVT: cerebral venous thrombosis; MRI: magnet resonance imaging; MRA: magnet resonance angiography; CTA: computed tomography angiography; PPV: positive predictive value; NPV: negative predictive value; PE: pulmonary embolism; DVT: deep vein thrombosis; ROC: Receiver Operating Characteristic curve; AUC: area under the curve

## Competing interests

The authors declare that they have no competing interests.

## Authors' contributions

The study was design by ES. CT and JA participated in conception and design. CT, ES, NS and RS carried out the data collection and drafted the manuscript. CT, ES and WP performed the statistical analyses. All authors were involved in the analysis and interpretation of the results. All authors revised the manuscript critically for important intellectual content and were involved in drafting the manuscript. All authors read and approved the final manuscript.

## Pre-publication history

The pre-publication history for this paper can be accessed here:

http://www.biomedcentral.com/1471-2377/11/69/prepub
